# The human genome as the common heritage of humanity

**DOI:** 10.3389/fgene.2023.1282515

**Published:** 2023-11-07

**Authors:** Faith Kabata, Donrich Thaldar

**Affiliations:** ^1^ School of Law, University of KwaZulu-Natal, Durban, South Africa; ^2^ Petrie-Flom Center for Health Law Policy, Biotechnology, and Bioethics, Harvard Law School, Cambridge, MA, United States

**Keywords:** human genome, common heritage of mankind, human genome project, human genomic research, human pangenome project, right to science

## Abstract

While debate on the international regulation of human genomic research remains unsettled, the Universal Declaration on the Human Genome and Human Rights, 1997 qualifies the human genome as “heritage of humankind” in a symbolic sense. Using document analysis this article assesses whether, how and to what extent the common heritage framework is relevant in regulation of human genomic research. The article traces the history of the Human Genome Project to reveal the international community’s race against privatization of the human genome and its resulting qualification as the common heritage of humanity. Further, it reviews the archival records of UNESCO’s International Bioethics Committee to discover the rationale for qualifying the human genome as common heritage of humankind. The article finds that the common heritage of mankind framework remains relevant to the application of the human genome at the collective level. However, the framework is at odds with the individual dimension of the human genome based on individual personality rights. The article thus argues that the right to benefit from scientific progress and its applications offers an alternative international regulatory framework for human genomic research.

## 1 Introduction

Traditionally, jurists, politicians and scholars have invoked distributive and preservationist aims to decide that certain natural and cultural assets outside national territorial limits should be regulated under the common heritage of mankind framework (Wolfrum, 1983). The UNESCO Universal Declaration on the Human Genome and Human Rights (UNESCO, 1997) affirms the human genome as the heritage of humanity. This places the human genome in the category of outer space, the moon and other celestial bodies, as well as the deep seabed. These are all common resources regulated under the common heritage of mankind framework.

The qualification of the human genome as the common heritage of humanity is, however, so much more. It is the outcome of a titanic battle by the international scientific community between open scientific inquiry versus proprietary science; open and freely accessible data versus proprietary databases; and common resources versus private property. The archival records of the drafting of the Universal Declaration of the Human Genome and Human Rights (Universal Declaration on the Human Genome and Human Rights, 1948) confirm that the qualification of the human genome as the heritage of humanity was coined to emphasise the “need for equitable pooling” of scientific knowledge of the human genome to benefit all of humankind (Committee of governmental experts for the finalization of the declaration on the human genome, 1997).

In this article, using documentary analysis, we demonstrate the relevance of the common heritage of mankind framework to the human genome, and review its adequacy for regulating human genomic research after the Human Genome Project (HGP). Several scholars have noted the common heritage of mankind framework (Gorove, 1972; Wolfrum, 1983; Joyner, 1986). Therefore, there is no need to rehash their detailed analysis, and we rather assess whether, how and to what extent the common heritage of mankind framework is relevant to regulation of research on the human genome using a two-pronged approach. First, we trace and demonstrate its enduring relevance to human genomics research. Second, reflecting on the adequacy of the framework for regulating human genomics research after the HGP, we explore the tension between the framework and the individual dimension of the human genome. In conclusion, we suggest the right to enjoy the benefits of scientific progress and its applications as an alternative international regulatory framework.

The article flows as follows. In part 2 we focus on the meaning of the human genome—based on the Universal Declaration on the Human Genome. In part 3, building on the discussion in part 2, we demonstrate the relevance of the qualification of the human genome as a common heritage of mankind through the history of the HGP and analysis of the drafting records of the Universal Declaration on the Human Genome. After the HGP, we demonstrate the relevance of the framework in relation to the pangenome, and highlight its inadequacies in the context of the individual human genome. In the concluding part 4, we discuss the right to enjoy the benefits of scientific progress and its applications as an alternative regulatory framework.

## 2 What is the human genome?

Despite the prominence and frequent appearance of the term ‘human genome’ in human genomic research, there is little conceptual clarity on the meaning of the human genome. Acknowledging the different usages of the term, we confine ourselves to the meaning assigned by international instruments. The Universal Declaration on the Human Genome and Human Rights (Universal Declaration on the Human Genome) (UNESCO, 1997) does not in the body of the Declaration define the human genome. Nonetheless, the Explanatory Notes attached to the Declaration (UNESCO General Conference, 1997) define the human genome as “both to the full set of genes of each individual—in the twin senses of genetic material (DNA molecules) and genetic information—and to the entire range of genes which constitute the human race”. Accordingly, the human genome is broadly the individual genome and the collective genome of the human species, and both genetic material and genetic data.

The broadness of this definition renders it meaningless. For instance, for the individual, his/her DNA and genetic information derived from the DNA constitute the human genome, while at the same time all the DNA and genetic information derived therefrom of the entire human species is the human genome. The human genome is thus the individual genome of each individual and also the collective genome of the entire human race. The UNESCO International Declaration on Human Genetic Data does not expressly mention the human genome, but elaborates on both genetic data and genetic material. These are referred to as biological samples, which are addressed as related concepts. This allusion to genetic data and biological samples embraces the individual dimension of the human genome.

Existing scholarship has offered some clarity. First, there is the human genome reference sequence which refers to a baseline map and a compound genome sequence of the human genome derived from the genetic data of several individuals that was generated by the HGP (National Human Genome Research Institute, 2022). The first draft of the human genome sequence was published in 2001, and later refined and updated in 2003 and 2010 (National Human Genome Research Institute, 2022). A complete sequence of the human reference genome, that which closed all the gaps, was released in 2022 (National Human Genome Research Institute, 2022). The definition of the human genome in the Universal Declaration on the Human Genome, the human genome reference sequence, represents the human genome at the collective level. This is because, as stated, it is a compound sequence generated from the genetic data of several individuals.

Second, on the individual dimension of the human genome, Thaldar et al in their analysis of the multidimensional legal nature of personal genomic sequence data offered some conceptual clarity (Thaldar, et al., 2022). The authors noted that personal genomic sequence data refers to individual genomic information that has been sequenced from DNA (Thaldar, et al., 2022).

In the next section we trace the application of the common heritage framework to the human genome and demonstrate its relevance in the regulation of research on the human genome.

## 3 The common heritage doctrine and regulation of the human genome

### 3.1 The human genome as “heritage of humankind”

The Universal Declaration on the Human Genome refers symbolically to the human genome as the heritage of humankind (UNESCO, 1997). While the term “heritage of humankind” had relatively little usage in international law, archival records indicate earlier drafts of the Universal Declaration on the Human Genome referred to the human genome as the common heritage of mankind. The International Bioethics Committee (IBC), in initial drafts, referred to the human genome as the common heritage of mankind, but the Committee of Governmental Experts dropped the term in favour of the “heritage of humankind in a symbolic sense” (International Bioethics Committee, 1996). Knoppers attributed these changes to differences among governmental representatives on the implication of the common heritage of mankind framework. Developing countries viewed the framework as allowing appropriation of the human genome by international companies, while the developed countries took a counter-position. They did not favour the communitarian aspect envisaged in the framework and were wary of state sovereignty, and thus preferred to protect the human genome at the individual level (Knoppers, 1999). According to the IBC, “heritage of mankind” was used to disabuse the notion that the human genome could be subjected to commercial appropriation (UNESCO General Conference, 1997).

Notwithstanding use of the term “heritage of humankind”, in terms of international regulatory mechanisms, the UNESCO Declaration on the Human Genome qualifies the human genome as the common heritage of mankind.

### 3.2 The human genome as the common heritage of humankind: the relevance

#### 3.2.1 Keeping the human genome in the public domain: race against privatisation

The qualification of the human genome as the common heritage of mankind traces to the HGP. The HGP is itself a story of many contrasts: international collaborative science versus national human genomic research initiatives; open access scientific inquiry versus private proprietary science; open freely accessible data versus proprietary databases; and the resulting human genome reference sequence as personal yet universal. These contrasts mirror the principles that embody the common heritage framework: national sovereignty versus international governance; sharing of benefits versus commercial principles; and common resource versus proprietary resource. These HGP contrasts sowed the seeds for the formulation of the 1996 Bermuda principles for free public access to the human sequence data and the qualification of the human genome as the common heritage of humanity.

The HGP was launched in 1990 as a 15-year international collaborative initiative involving a group of scientists from the United States, United Kingdom, France, Germany, Japan, Canada and China known as the International Human Genome Sequencing Consortium (Consortium, International Human Genome Sequencing, 2001). The primary objective of the HGP was to map, locate and sequence the human genome, with smaller affiliated projects involving the sequencing of the model genomes of the worm, fruit fly, yeast and mouse (Consortium, International Human Genome Sequencing, 2001). The primary goal was to generate a reference sequence of the human genome. By 1995, the HGP completed the first phase: the construction of the genetic and physical maps of the human genome (Consortium, International Human Genome Sequencing, 2001). The second phase was completed in 2001, and was marked by the release of the first draft of the human reference sequence of the human genome in February 2001 (Consortium, International Human Genome Sequencing, 2001). Updated drafts of the human reference sequence were released in 2003 and 2010, and the final complete version in 2022 (National Human Genome Research Institute, 2022). The results of the human sequence indicate that human beings are 99.9% similar, with the 0.1% accounting for genetic variance among individuals (Consortium, International Human Genome Sequencing, 2001).

However, the above account is the less debated part of the HGP story and does not account for its main legacies—the Bermuda principles for data sharing and qualification of the human genome as the common heritage of humanity. First, it should be noted that national and private enterprise endeavours to sequence the human genome predate the 1990 launch of the HGP. Prior national initiatives included: the USA’s human genome research under the Office of the Human Genome Research; the 1981 Japan’s Science and Technology Project which aimed to convert genome sequencing into a large-scale project; the French Centre d’Etude du Polymorphisme Humain, established in 1994 with the goal of creating genetic maps of all chromosomes in the human genome; and the UK’s Medical Research Council set up in 1988 to coordinate mapping and sequencing of the human genome (Raggio, 2002). Private enterprise endeavours included the Genome Corporation which in 1987 announced plans to sequence the human genome and to commercialise the data (Raggio, 2002). While the national human genomic research interests were mediated in the HGP, threats to commercialise the human sequence data by private enterprise endeavours overshadowed the HGP throughout its life.

Controversy over the public or private nature of the human genome first arose at the beginning of the HGP in 1991 when the National Institutes of Health (NIH) filed 337 patent applications for gene fragments sequenced by Venter, who was then a scientist at the NIH (Eisenberg and Nelson, 2002). The international collaborators of the HGP considered these patent applications by the NIH to be in contradistinction with the primary objectives of the HGP—to sequence the heritage of humanity (Eisenberg and Nelson, 2002). For instance, the French National Consultative Committee on Ethics condemned the patent applications and indicated that the information contained in the human genome was part of common heritage of humanity, and hence could not be monopolised (Dworkin, 1997). In 1992, Venter left the NIH to set up the non-profit Institute for Genomic Research, which was affiliated to the private firm Human Genome Sciences (Mukherjee, 2016; Cook-Deegan, et al., 2017). Human Genome Sciences backed Venter’s earlier work on expressed sequence tags and established proprietary databases on these gene sequences which locked out access to researchers in academic institutions (Eisenberg and Nelson, 2002; Cook-Deegan, et al., 2017).

These concerns over the “gold rush” to privatise the human genome were part of the agenda of the 1996 Bermuda meeting captured as patenting of the human genome and data sharing (Cook-Deegan, et al., 2017). The session on data sharing noted: “The fact is that we’d come to realize that the genomic sequence we are producing and dealing with is more than a commodity. It is the essence of biological heritage, the instruction book of living things. The only reasonable way of dealing with the human genome sequence is to say that it belongs to us all—it is the common heritage of humankind” (Bradley, 2005). The final statement from the session read: “It was agreed that all human genomic sequence information generated by centers for large-scale human sequencing, should be freely available and in the public domain, in order to encourage further research and development, and to maximise its benefit to society” (Cook-Deegan, et al., 2017). This data sharing agreement on daily online release of human sequences under the HGP is referred to as the Bermuda principles for data sharing (Cook-Deegan, et al., 2017).

Even after the Bermuda principles, the race to keep the human genome from private enterprise was far from over. In 1998, as the HGP was embarking on sequencing the human genome, Venter broke away from the HGP and established Celera Genomics, a new private company. Under Celera Genomics, Venter announced plans to sequence the human genome using a faster methodology and more cheaply, and aimed to complete the sequencing within three years—four years ahead of the HGP—and establish commercial proprietary databases (García-Sancho, et al., 2022). In addition, contrary to the Bermuda principles on daily data release, Venter indicated that Celera Genomics would release data every 3 months (Eisenberg and Nelson, 2002; Jasny, 2013). Challenged by the HGP that quarterly release was contrary to the Bermuda principles, Venter retorted: “[w]e are a company … We do not have to release the data at all. But if you think about it, quarterly is a lot closer to nightly than it is to never” (Jasny, 2013).

This announcement by Celera Genomics began the most polarising race between the HGP and private enterprise. The implication of the announcement was that it put the utility of the HGP into question, as it suggested that sequencing could be achieved faster and more cheaply and threatened to forever put the human genome in the hands of private enterprise. In response, at the technical level, the HGP revised its strategy: it requested more funding to speed up the sequencing and importantly shifted the priority to producing a ‘rough draft’ of the human genome by 2000 rather than a complete sequence in 2005 (Eisenberg and Nelson, 2002; Raggio, 2002). At the political level, the United States government pre-empted the race by issuing a joint statement by the US President and United Kingdom Prime Minister in March 2000, which declared that the human sequence DNA should be made freely available to all scientists across the globe (Raggio, 2002). In June 2000, the US President and United Kingdom Prime Minister also presided over a joint release by the HGP and Celera Genomics of the ‘rough draft’ of the human genome reference sequence (Consortium, International Human Genome Sequencing, 2001), which effectively ended ‘the race’. In line with the Bermuda principles, the HGP released its data on the human reference sequence in *Nature* in February 2001, while Celera Genomics published its sequence in *Science* a day later, although with some restrictions to full access. However, contentions abound on the quality of the human genome reference sequence released by Celera Genomics based on the methodology of sequencing and claims that it benefited from the HGP data to generate its own human reference sequence (Waterston, et al., 2002).

The enduring legacy of the HGP was keeping the human genome in the public domain through the daily data release policies and the common heritage of humanity qualification. A key observation from the foregoing is that for the international sequencing community in the HGP, the essence of the human genome as the common heritage of humankind was to protect and promote freedom of research in the scientific community. The race was thus between open and free scientific inquiry versus private proprietary science, and between open and freely accessible data versus proprietary databases.

UNESCO waged an equivalent race to keep the human genome in the public domain. In 1997, the IBC, comprising scientists and legal scholars, affirmed the human genome as the common heritage of humanity in the Universal Declaration on the Human Genome. In at least two ways UNESCO’s qualification of the human genome as the common heritage of humanity coincided with events at the HGP. First, Knoppers alluded to the fact that there were already proposals as far back as 1991 to declare the human genome, at the collective level, as the common heritage of humanity (Knoppers, 1999). As noted earlier, the initial attempt at national privatisation of the human genome was in 1991 when the NIH filed for patents for gene fragments of brain cells. The concern then was both for scientists and for other states that had foregone their national genomic research initiatives for the collaborative HGP. Second, the Universal Declaration on the Human Genome drew from the spirit of the 1996 Bermuda principles. As alluded to earlier, the Bermuda meeting stated that “the human genome belongs to us all”, and hence it is plausible to link the UNESCO affirmation with the position taken by the international sequencing community.

In addition, archival records of the IBC’s discussions reveal that UNESCO was concerned with national appropriation of the human genome by developed countries. This concern was well founded. Besides the USA’s 1991 attempt to patent gene fragments, the composition of countries that participated in the HGP validated this concern. According to the HGP architects, the HGP was founded on the principle of inclusivity as the genome was the common heritage of all humankind, and thus any nation could participate by opening mapping and sequencing centres (Waterston, et al., 2002). The HGP participating countries were the United States, United Kingdom, Canada, France, Germany, Japan, China and the European Community—the most developed countries with the technological infrastructure and human capacity. Illustratively, the then Chair of IBC, Mohammed Bedjaoui, stated that, “any advance in knowledge on the human genome must benefit mankind as a whole … a common heritage regime is mindful of the inequalities in the development of various regions in the world” (Kuppuswamy, 2009). Therefore, UNESCO was also waging a war against bio-colonialism: the appropriation of the human genome by developed countries without any benefits accruing to developing countries. The common heritage of humankind framework was deployed for regulation of the human genome as a resource belonging to all of humanity. Significantly, while the HGP’s main motivation for qualifying the human genome as the common heritage of humanity was freedom of research, UNESCO privileged equity, social justice, and benefit sharing from the Global North-South perspectives. These ideals find expression in the provisions of the Universal Declaration on the Human Genome (UNESCO, 1997) on the regulation of the human genome.

#### 3.2.2 The human genome as the common heritage of mankind

The moon, outer space and the deep sea bed and ocean floor are all considered common resources, which are outside territorial limits of national jurisdiction and from which no person should be excluded and for which there should be no individual or government appropriation. However, they should rather be publicly regulated to distribute the benefits and preserve them for future generations. Consequently, these common resources are internationally regulated under the common heritage of mankind framework. Significantly, unlike the common property doctrine, the common heritage of mankind framework requires that all manage the resources and share in the benefits, including those who do not participate in the exploitation of the resources (Noyes, 2011). What then does it mean for the human genome as the common heritage of humanity?

As discussed in the foregoing, HGP’s two principles on human genome sequencing embedded the common heritage of humanity doctrine. First, the collaborative nature of the HGP was informed by the universal nature of the human genome sequence as the common heritage of humanity transcending national territorial limits (Waterston, et al., 2002). Second, the principle of unrestricted data release as articulated in the 1996 Bermuda principles was founded on the idea that the human genome sequence ‘belongs to us all’, and that it is a common resource (Waterston, et al., 2002). Furthermore, the HGP was conceived as a universal project to generate the human reference sequence, and therefore it sequenced the individual DNA of a diverse group of anonymous individuals who maintained no further association with the data (Contreras and Knoppers, 2018a). Taken together, the conceptualisation and nature of the HGP pointed to humanity’s collective ownership of the human reference sequence. Knoppers and Beauvais have noted that given the nature of the HGP, the human reference sequence was a common resource for humanity that could not be controlled by an individual or private enterprises or government (Knoppers and Beauvais, 2021). The Universal Declaration on the Human Genome defines the human genome as both the individual and collective genome. Records of the drafting sessions of the IBC reveal that in applying the common heritage doctrine in the field of genetics, the IBC indicated that the aim was “safeguarding the integrity of the human species” (International Bioethics Committee, 1996).

A consideration of the above context reveals that the common heritage of mankind doctrine applies to the human genome at the collective level: the human species level. The individual genome thus does not qualify as the common heritage of mankind. Knoppers has alluded to the application of the common heritage of mankind framework to the human genome at the human species level (Knoppers, 2005; Knoppers and Joly, 2007). Applying the common heritage of humankind doctrine to the human genome, two questions arise. What common resources does the doctrine apply to? And what are its elements? On the first question, the doctrine applies to areas outside the territorial limits of states and to the natural resources in those areas (Noyes, 2011). The human genome at the human species level refers to the human reference sequence of humanity, which embodies the universality of the human species unbounded by state territorial limits. It thus qualifies as common resources beyond state territorial limits. On the second question on the elements of the doctrine, although unsettled, consensus exists on the following: (i) a ban on the acquisition of or exercise of sovereignty over the resources; (ii) rights over the resources vest in humankind; (iii) equitable sharing of benefits derived from exploitation of the resource, with particular consideration of the needs of developing states; (iv) common management of the resources; (v) use of the resources for peaceful purposes; and (vi) protection of the environment (Wolfrum, 1983; Noyes, 2011).

Reflecting on the human genome at the collective level, the first four elements noted above are important. On the ban on acquisition of or exercise of sovereignty, the HGP by its very nature, conceptualisation and coordination, as discussed earlier, ensured that the human reference sequence was not appropriated by national states, individuals or corporations. The open and free release of the human reference sequence put the resource in the hands of humanity, from which no entity could be excluded and no entity could claim exclusive control. It then follows that the human reference sequence belongs to all of humanity and humanity has rights over its use and disposal. On the element of sharing of the benefits derived from exploitation of the resources, Wolfrum and Noyes have pointed out its controversial nature (Wolfrum, 1983; Noyes, 2011). The controversy on sharing of the benefits mainly arises from the assertion that this includes preferential treatment for developing states (Noyes, 2011). Discussing the application of the doctrine to the seabed and ocean floor, Wolfrum argued that since all states participate equally, directly or indirectly, in the exploitation of the seabed minerals, the idea of preferential treatment was discarded (Wolfrum, 1983). In addition, a question may be asked about the scope of the shared benefits, and whether it includes the results of scientific research. Viewed from the actual implementation of the common heritage of mankind framework in the law of the sea regime, scientific research results fall within the scope of shared benefits, while preferential treatment in the distribution of benefits for developing states was subjected to market principles (United Nations, 1994). Finally, common management of the resource is anchored on the idea that humankind is vested with rights over control of the resources, where an international entity or forms of cooperative arrangements would be required to act at the instance of humankind (Noyes, 2011). In relation to the human genome, the scientific results of the human reference sequence are available for all and to that extent the element of benefit sharing seems to hold. However, the human sequence as generated by the HGP is a reference map resource, and any health benefits that accrue to humankind would be the result of further scientific research.

#### 3.2.3 After the HGP: the common heritage of humankind framework and the human pangenome reference project

Beyond the HGP, does the common heritage framework have any relevance? As demonstrated above, the main legacies of the HGP were the human reference sequence and the Bermuda principles for data sharing. After the HGP, scholarship has identified concerns in human genomic research. An editorial in *Nature* in February 2021 identified the enduring concerns as: ethical and legal issues such as privacy and consent; representation of both data contributors and users; and challenges in implementation of access to genome data (Nature Editorials, 2021). The editorial provided further elaboration of the concerns as: data collection from the participants; data deposits in publicly accessible and approved databases; and data access (Nature Editorials, 2021). Similarly, Knoppers, Contreras and Cook-Deegan et al. note that after the HGP, ethical, legal and technical issues such as the protection of individual data and researchers’ publication priority have chipped away the expanse of data sharing envisioned in the Bermuda principles (Contreras, 2011; Cook-Deegan, et al., 2017; Contreras and Knoppers, 2018b).

In relation to the diversity deficit in the human reference sequence, the human pangenome reference project was initiated in 2019 under the Human Pangenome Reference Consortium and is expected to sequence, assemble and freely share the human pangenome reference which will correctly reflect the diversity of the human species (Miga and Wang, 2021; Liao, 2023). The human pangenome reference project is similar to the HGP in that it is an international collaborative science initiative and involves sequencing DNA from 350 individuals of diverse ethnic backgrounds to create a baseline reference sequence (Miga and Wang, 2021). It is therefore a community resource project aimed at generating reference data for human genomic research. The first draft of the human pangenome reference sequence was released in May 2023 and consists of 47 sequenced and assembled diverse individual human genomes which feature the diversity within the human species (Liao, 2023). The Human Pangenome Reference Consortium will increase the number of individual human genomes sequenced and assembled to 350 individuals by 2024 (Liao, 2023). Even then, the pangenome project is not without criticism as to its diversity and inclusiveness. It has been pointed out that the pangenome project appears focused on numerics without proper consideration of the communities and nations to collaborate with in order to address the diversity deficit (Cho, et al., 2023). Unlike the human reference sequence under the HGP, with the pangenome sequence the researchers indicated that consent was obtained from 47 individuals for the release of the draft pangenome human sequence (Liao, 2023). The implicit question is whether the common heritage of mankind framework is relevant to the human pangenome sequence.

While there has been a narrowing of the original scope of data sharing under the Bermuda principles, Knoppers and Contreras have noted that the Bermuda principles apply to community resource projects, that is research aimed at generating data for use by the scientific research community (Contreras and Knoppers, 2018a). This position is also affirmed by Cook-Deegan et al., who noted that the data sharing obligations of research projects aimed at generating community resources remained governed by the Bermuda principles, despite a watering down of obligations for hypothesis-focused research (Cook-Deegan, et al., 2017). Therefore, given that the human pangenome sequence project aims to generate data for the scientific community, drawing from the HGP approach, the common heritage framework can apply to the human pangenome reference at the collective level—the human species level.

On the future of the application of the common heritage framework in human genomic research, it is notable that up to now under international law the framework has been implemented only in the law of the sea regime. And, as alluded to earlier, what was operationalised and implemented is a diluted version of the framework, in particular with regard to the sharing of benefits. Noyes, while discussing the application of the common heritage of mankind framework to other common resources besides the seabed minerals, noted that sharing of benefits and common resource management are the most contested elements, because of the finite nature of the resources. Furthermore, the likelihood of extending the framework to other common resources would require redefining the elements of the framework (Noyes, 2011).

Even beyond these general contestations, in human genomics research the conceptual underpinnings of the common heritage of mankind framework present important considerations. These include: it is associated with natural resources, leading to the question of whether the human genome, in particular in its individual dimension, can be considered a natural resource; human genomic data is infinite, and hence the problem of a depletion of resources fear that characterizes the common heritage of mankind framework does not apply; and the preservation ethic aimed at conserving the resources, particularly given that the human genome evolves. These considerations resonate with the ethical, legal and social concerns identified above in human genomic research after HGP.

We now explore these inadequacies of the common heritage of mankind framework from the individual dimension of the human genome.

### 3.3 Common heritage of mankind framework, individual rights and species preservation

The underpinnings of the common heritage of mankind framework appear to be at odds with the individual dimension of the human genome. First, the notion of a common resource under common heritage raises the following questions: Can individuals, genes and genetic information in the individual genome be considered a common resource; and can the common heritage doctrine be reconciled with individual personality and property rights inherent in genomic resources? Second, the preservationist bias that underpins the common heritage doctrine also raises questions about individual rights such as the right to health, life and to enjoy the benefits of scientific progress and its applications.

#### 3.3.1 Individual personality rights and the “heritage of species”

Thaldar et al., in their analysis of the multidimensional legal nature of personal genomic sequence data, identified individual personality rights in the data as: personal integrity, respect of a person’s identity and informational privacy (Thaldar, et al., 2022). The authors also noted that personality rights attach to the individual and cannot be lost. Furthermore, individual personality rights take precedence over any property rights or claims that may be made in relation to the data (Thaldar, et al., 2022). The right to informational privacy entails control over use, access and processing of personal genomic sequence data (Thaldar, et al., 2022). Tied to this is the notion of informational self-determination which gives the individual sovereignty and control over their data (Hummel, et al., 2019). The right to personal identity entails the right of an individual to construct a life narrative of themselves based on what they consider important (De Andrade, 2010). Implicit in this right is the right to individual data sovereignty by controlling its use and processing.

As discussed, the common heritage of mankind framework regulates common resources and is relevant for the human genome in its collective dimension. However, in relation to the individual human genome, as Thaldar et al have noted, individual personality rights take precedence (Thaldar, et al., 2022). The common heritage of mankind framework based on its patrimonial foundations cannot be reconciled with individual personality rights that arise in relation to the individual dimension of the human genome (De Andrade, 2010). The UNESCO Declaration on Human Genetic Data embraces the individual personality rights as it refers consent for collection and use of genetic data and biological samples to the individual (UNESCO, 2003). The Declaration on Human Genetic Data is proclamatory, and thus has no legally binding obligations on states. Rather, it defers the protection of individual personality rights to states.

Flowing from the Declaration on Human Genetic Data, states have put in place mechanisms for the protection of individual personality, including privacy, informational self-determination and respect for personal identity in the context of human genomic research. Equally, states have an obligation under the Covenant on Economic, Social and Cultural Rights to guarantee the right to enjoyment of the benefits of scientific progress and its applications. This takes a cue from the indivisibility of human rights, individual personality rights and the right to the benefit of scientific progress and its applications which are interdependent and interconnected, and no right should take precedence over another. Knoppers and Beauvais noted that enjoyment of the right to the benefits of scientific progress and its applications is premised on data sharing, which invokes individual personality rights as individuals exercise informational self-determination by deciding which data to share or control (Knoppers and Beauvais, 2021). Therefore, states in their obligations to guarantee the right to enjoyment of the benefits of scientific progress and its applications must put in place a regulatory framework that ensures respect for privacy and individual genetic data control, including the right to one’s personal identity based on their genetic data.

#### 3.3.2 Individual property rights

As noted earlier, Thaldar et al. noted that personal genomic sequence data can be owned privately, can be public property and also can be common resources under the common heritage of mankind framework (Thaldar, et al., 2022). In addition, the authors posited that since personal genomic sequence data is generated from DNA sequencing, a number of entities, including the research institutions and funders, may lay a claim of ownership (Thaldar, et al., 2022). Furthermore, they suggested that an entity can acquire ownership of personal genomic sequence data through appropriation if it has effective control of the data as a digital object (Thaldar, et al., 2022). However, ownership rights in personal genomic sequence data are subjected to the individual personality rights of the data subject (Thaldar, et al., 2022). In essence, in relation to the individual human genome, the individual has certain entitlements in their personal genomic sequence data, based on personality rights that trump ownership rights.

Therefore, in relation to the individual human genome, while the person or entity in control of the personal genomic sequence data may claim ownership, the personality rights of the research participant limit such ownership. In the context of exercise of individual personality rights in genomic research, the right to informational self-determination would entitle the research participant to control use of and access to their data.

#### 3.3.3 Preservation of the human genome: safeguarding species integrity and the natural evolution bias

In qualifying the human genome as the common heritage of mankind framework, the IBC was motivated to “safeguarding the integrity of the human species” (International Bioethics Committee, 1996). In addition, the IBC took note of the natural evolution of the human genome ascribing to the idea that natural evolution is responsible for the human genome (UNESCO, 1997). The HGP sequence of the human genome revealed that 50% of the human genes are similar to the genes of other model organisms sequenced such as the worm, fruit fly and mouse, thus displacing any special expectations on the human genome (Goes, 2016). This questioned the claim of specialty of the human species and the idea of the species barrier. Harris has argued that the claim of integrity of the human species does not hold. First, he posited that claims of maintaining a species barrier between the human person and non-humans overlook the fact that through diet, drugs, vaccines and xenotransplantation, exchange of biological material often occurs between the human person and animals (Harris, 2011). Harris observed that these instances which involve mixing of the biological matter from animals to the human person are not frowned upon as an interference with the purity of human species (Harris, 2011). Based on this, he questioned barring scientific interventions in the human genome to safeguard the integrity of the human species, while the above practices that involve mixing of human and non-human genes are acceptable. Second, Harris noted that based on evolution theory, the genetic makeup of the human person includes genes from all other creatures that the person has over time evolved from (Harris, 2011). Based on the evolution process, the argument on purity of the human species is flawed and, therefore, there is no basis for safeguarding the integrity of the human species.

Similarly, Knoppers and Joly noted that the idea of safeguarding the integrity of the human species is based on the preference for maintenance of the natural order over scientific interventions, which are viewed as interfering with the purity of the human species (Knoppers and Joly, 2007). They argued that appeals to purity of the human species should not be a justification for human persons not to benefit from scientific progress (Knoppers and Joly, 2007). On the natural order, Knoppers and Joly have called for a reconceptualisation of what is considered natural and a shift away from viewing the human person with the naturalism lens (Knoppers and Joly, 2007). According to Harris, the preservationist ethic embedded in the common heritage of mankind framework is flawed as it ignores that natural human reproduction already changes the human genome, and therefore the human genome cannot be considered as frozen in time (Harris, 2015).

Harris also argued against UNESCO’s bias towards the natural order, noting that natural evolution is slow and does not guarantee improvement of the human species, while scientific progress would guarantee improvements in the quality of health and life of humankind (Harris, 2015). He pointed out that the bias towards natural evolution is premised on the wrong assumptions that the natural order is good and not capable of improvement and that natural evolution enhances the human genome for the better (Harris and Søren, 2002). Ultimately, the bias to the natural order impedes the enjoyment of various individual rights as individuals cannot benefit from scientific progress and also exercise informational self-determination.

A common theme in the arguments of Harris and Knoppers and Joly is the effect of the absolute construction of individual personality rights such as privacy, autonomy and human dignity on the right to enjoy the benefits of scientific progress and its applications. Knoppers and Joly have questioned the invocation of human dignity concerns as a bar to application of scientific inventions to human beings (Knoppers and Joly, 2007). The authors have noted that individuals enjoy human dignity by virtue of personhood, and that personhood is not diminished by application of scientific interventions on the human body (Knoppers and Joly, 2007). Supporting this proposition, Harris drew from scientific experiments in the 1990s which involved interventions from animals to humans—noting that mixing of genes does not change the characteristics of a species (Harris, 2011). Thus, personhood and the human dignity that attaches to personhood is not lost through scientific interventions. In the same line of argument on human dignity, Jordaan, writing on stem cell research in the Brüstle case before the European Court of Justice, observed that human dignity attaches to human beings, but is often deployed as a mask for abstract claims anchored in morality (Jordaan, 2017). Knoppers and Joly and Jordaan raised related concerns on how human dignity should be deployed in relation to genomic research, should it promote a conception of dignity that attaches to abstract humanity or to real personhood. For Knoppers and Joly, the question is whether it should be invoked to promote species purity rather than to advance the right to health and life. Jordaan criticised the invocation of human dignity to abstract embryos (which in many jurisdictions are not considered as human beings), rather than invoking human dignity to advance the right to health. Generally, on human rights, Harris has taken a more blunt view and posited that the concept of humanness should give primacy to the powers and capacities that improve the quality of existence of the human person, rather than deploying human rights as an obstacle to scientific interventions on the human genome (Harris, 2011).

In sum, as noted earlier, human rights are indivisible, and enjoyment of one set of rights does not curtail the enjoyment of other rights. The individual personality rights and the right to enjoy the benefits of scientific progress and its applications are not absolute. There is therefore room for proportionality in the exercise of each set of rights to ensure that there is no hierarchy in rights but instead an interdependence of rights. In addition, on human dignity in human genomic research, the notion of human dignity as a mask for morality and the natural order should be discarded.

## 4 Conclusion

The article demonstrates the relevance of the common heritage of mankind framework to the human reference sequence and also to the pangenome reference sequence at the collective level. The qualification of the human reference sequence as a common resource open and freely accessible to all humanity, provided a framework for collaboration in sharing of genomic data within the scientific community, hence facilitating the realisation of the right to freedom of research. As noted earlier, the enduring concerns in human genomic research after the HGP are: data collection from participants; depositing data in publicly accessible and approved databases; and data access (Nature Editorials, 2021). And as demonstrated by the article, the common heritage framework is at odds with the individual rights, putting into question the extent to which the framework is able to protect the rights and interests of the diverse stakeholders in human genomic research. Thorogood et al. identified the rights and interests of the different stakeholders as: recognition of data generators; interests of data users in accessing data; rights of participants to benefit from the research and to protection of their data rights (Thorogood, et al., 2015). The authors proposed that the international human rights law framework is best suited for bringing together the multiple interests and rights involved as it is universal and transcends state borders, it has legal and political binding force and it imposes obligations beyond the scientific community to states, private actors and protects the rights of individuals (Thorogood, et al., 2015). Specifically, the authors discussed the right to enjoy the benefits of scientific progress and its applications (right to science) as a possible framework for human genomic research data sharing (Thorogood, et al., 2015).

Drawing from these insights, the article highlights the relevance of the right to science as an alternative framework for sharing of genomic data. The UN Committee on Economic, Social and Cultural Rights elaborated on the right to science in General Comment No. 25 (CESCR, 2020). In the context of human genomic research data sharing, the right to science imposes obligations on states and the international community to safeguard the rights of data generators, data users and research participants. In relation to recognition of data generators, the right requires states to ensure that contractual arrangements provide appropriate crediting and acknowledgment of the contributions of scientific researchers to research outcomes as a consequence of the right to freedom of research. For data users, states have a duty to facilitate international cooperation that enables researchers to freely share data and collaborate internationally. For research participants, the right imposes an obligation on states to ensure access of their population to health benefits that accrue from human genomic research, including fostering a positive balance with intellectual property, as well as adopt normative standards for the protection of privacy and data rights and human dignity (CESCR, 2020).

For actualization of the right, beyond states’ implementation of the above discussed obligations, there is a need to reframe the perception of the right. On implementation of state obligations, states should put in place regulatory frameworks that ensure the respect and protection of the rights of individual genomic data in the context of human genomics research. An additional obligation is to conduct public education to promote participation of individuals in the advancement of science, in particular through data sharing. On reframing the perception of the right to enjoy the benefits of scientific progress and its applications, the idea is to relook at implementation of the right in relation to individual personality rights that seek to protect the human person as an autonomous individual. Practices in human genomics research have mainly focused on protecting individuals from harm that would be associated with research, and hence the dominance of rights protecting privacy, human dignity, informational self-determination and identity. However, the aspect of the benefits that accrue from science and its applications appears to be neglected. A reframing of the right to emphasise the benefits dimension will also result in a shift from the absolute construction of individual personality rights in human genomic research to a construction that allows for their interdependence with the right to enjoy the benefits of scientific progress and its applications. In part, there is also a need for appreciation of the right to science as a collective endeavour, in that in genomics research the benefits of science result from human solidarity rather than absolute notions of individual autonomy. Finally, in relation to human dignity, while it is a fluid concept, given its obsessive repetition in the primary instruments regulating the human genome (UNESCO, 1997; UNESCO, 2005), there is room to define its scope and contours in relation to human genomic research. Currently, as noted above, human dignity has been invoked to mask claims of morality, instead of invoking it to promote enhancement of the human genome that promotes human dignity of individuals and humanity as a whole.

## Data Availability

The original contributions presented in the study are included in the article/supplementary material, and further inquiries can be directed to the corresponding author.
